# The effectiveness and cost evaluation of pain exposure physical therapy and conventional therapy in patients with complex regional pain syndrome type 1. Rationale and design of a randomized controlled trial

**DOI:** 10.1186/1471-2474-13-58

**Published:** 2012-04-19

**Authors:** Karlijn J Barnhoorn, Rob A B Oostendorp, Robert T M van Dongen, Frank P Klomp, Han Samwel, Gert Jan van der Wilt, Eddy Adang, Hans Groenewoud, Henk van de Meent, Jan Paul M Frölke

**Affiliations:** 1 Department of surgery 690, Radboud University Nijmegen Medical Centre, Nijmegen, Postbox 9101, 6500, HB, The Netherlands

## Abstract

**Background:**

Pain Exposure Physical Therapy is a new treatment option for patients with Complex Regional Pain Syndrome type 1. It has been evaluated in retrospective as well as in prospective studies and proven to be safe and possibly effective. This indicates that Pain Exposure Physical Therapy is now ready for clinical evaluation. The results of an earlier performed pilot study with an n = 1 design, in which 20 patients with Complex Regional Pain Syndrome type 1 were treated with Pain Exposure Physical Therapy, were used for the design and power calculation of the present study.

After completion and evaluation of this phase III study, a multi-centre implementation study will be conducted.

The aim of this study is to determine whether Pain Exposure Physical Therapy can improve functional outcomes in patients with Complex Regional Pain Syndrome type 1.

**Methods/design:**

This study is designed as a single-blinded, randomized clinical trial. 62 patients will be randomized with a follow-up of 9 months to demonstrate the expected treatment effect. Complex Regional Pain Syndrome type 1 is diagnosed in accordance with the Bruehl/International Association for the Study of Pain criteria. Conventional therapy in accordance with the Dutch guideline will be compared with Pain Exposure Physical Therapy. Primary outcome measure is the Impairment level SumScore, restricted version.

**Discussion:**

This is the first randomized controlled study with single blinding that has ever been planned in patients with Complex Regional Pain Syndrome type 1 and does not focus on a single aspect of the pain syndrome but compares treatment strategies based on completely different pathophysiological and cognitive theories.

**Trial registration:**

Clinical trials NCT00817128; National Trial Register NTR2090

## Background

Complex Regional Pain Syndrome type 1 (CRPS-1) or reflex sympathetic dystrophy (RSD) is a chronic progressive condition of an extremity characterized by a variety of autonomic, sensory, motor and vasomotor symptoms, usually following injury.

It describes a variety of painful conditions following injury which appears regionally having a distal predominance of abnormal findings, exceeding in both magnitude and duration the expected clinical course of the inciting event [1].

It often results in impairments of neuromusculoskeletal and movement-related functions, sensory functions and pain, activity limitations and participation restrictions. It shows a variable progression in the course of time.

Clinical evaluation and diagnosis of CRPS-1 are based on clinical assessment of signs and symptoms, in accordance with predetermined sets of diagnostic criteria [1].

Conventional therapy, consisting of pharmacological pain management and pain avoidance in functional management, has shown disappointing results in pain control and disabilities, and often leads to inactivity and social disintegration. Considerable impairments are still present over eight years after first CRPS-1 diagnosis [2].

Pain Exposure Physical Therapy (PEPT) is a new and promising therapeutic approach [3,4]. No prospective, randomized studies have yet been performed to evaluate its effectiveness and efficacy compared to the conventional therapy.

The CRPS outpatient department of the Radboud University Nijmegen Medical Centre has a long history regarding treatment and research on CRPS-1 [2-7].

### Magnitude of the problem

The incidence of CRPS-1 in the Netherlands is estimated at around 26.2:100,000 person years and females are 3.4 times more effected than males [8]. About 22% of patients show long-lasting symptoms and signs [2]. About 30% of CRPS-1 patients completely omit their work during more than one year. [9].

A vast proportion of CRPS-1 patients needs major adaptations of their homes to be able to function with their activity limitations and participation restrictions.

### Treatment of CRPS-1

#### Conventional therapy

Current conventional therapy for CRPS-1 has been consolidated in the Dutch national clinical guideline of 2006 [10]. It is focused on the relief of symptoms and pain with pharmacological treatment and immobilization of the affected limb, supported by physical therapy (e.g. by increasing control of pain, improving skills and optimizing coping with CRPS-1) and occupational therapy (e.g. by improving functional abilities and independence in activities of daily living) [6]. It results in approximately 50% reduction of disability and pain in patients with CRPS-1.

Pharmacological treatment consists of analgesics in a step-up procedure in accordance with the WHO’s pain ladder. Dimethylsulphoxide 50% ointment (DMSO) is prescribed as radical scavenger for initial treatment. In cases presenting with allodynia or hyperalgesia, gabapentine, amitryptilin or carbamazepine may be indicated. Dystonia, myoclonia and muscle spasms may be treated with baclofen, diazepam or clonazepam. Vasodilating drugs like verapamil, ketensin and pentoxiphyllin can be prescribed for patients presenting with cold skin. In persistent cold CRPS-1, thoracic sympathectomy can be considered as a treatment option [10].

#### Pain exposure physical therapy

PEPT is a functional form of physical therapy and consists of a progressive-loading exercise program and management of pain-avoidance behaviour without the use of specific CRPS-1 medication or analgesics. It is based on the assumption that behavioural and psychological factors can exacerbate pain and dysfunction and might help maintain the condition. Patients denominate clear treatment goals in the domains of activities and participation.

PEPT aims to decrease kinesiophobia, pain behaviour and learned non-use [11], and increase self-confidence in the patients’ own physical possibilities. Living without adaptations or living independently from caregivers, returning to work and employment, and restarting domestic life, self-care, mobility, hobbies and sports in a short time are the main treatment goals. Pain relief itself is not a primary treatment goal, and patients are informed that an increase in pain during or after the exercises and activities might occur. Patients are reassured that an increase in pain is not a sign of injury or tissue damage. In this respect, all conscious and unconscious signs of catastrophizing and kinesiophobic behaviour are specified and talked through with the patient and partner. If, despite explanation, doubt remains about the treatment content or when patients are not motivated to act upon instructions of the therapists, the treatment will be ceased.

The treatment consists of progressive-loading exercises and desensitization beyond the patients’ pain limits. To decrease the enhanced skin sensitivity for touch and pressure, desensitization is carried out using self-massage and forced use of the affected arm or leg in daily activities. The progressive-loading exercises are tailored and focused on specific body functions using standard techniques in regular physical therapy, including passive and active exercises to mobilize joints and muscle stretching. During progressive loading, the physical therapists act mainly as instructors, rewarding functional progression and providing schedules for exercises and activities at home. Complaining about pain is discouraged and it is no longer a subject of debate or a reason to reduce the treatment intensity. Partly due to a limited number of five sessions, PEPT is a very low-cost approach from micro- as well as macro-economic point of view.

### Rationales

The pathophysiology of CRPS-1 is not well understood. Most of the studies that have been carried out are observational in nature, with small numbers of patients taking part and, in most cases, without a control group. The conclusions of the studies are not always direct observations, but tend to be interpretations of the observations made by the investigators. A distinction can be drawn between peripheral afferent, efferent and central mechanisms involved in the onset and continuance of CRPS-1. So far, no clear explanation has been found of how these various mechanisms are related to one another. Since no randomized controlled trial have been published yet to validate the different treatment strategies in patients with CRPS-1, the rationale for a specific treatment is considered to be sufficiently convincing to perform individual treatments in patients.

#### Conventional therapy

It seems that a number of peripheral afferent mechanisms may be involved in the pathophysiology of CRPS-1. Indications point to an inflammatory process, a neuroinflammatory process and tissue hypoxia. Anatomical changes have also been seen in CRPS-1 patients. If a (neuro)inflammatory process is involved, then immunological predisposition may be relevant. There are indications that immunological acquired and/or genetic susceptibility may be involved in the onset of CRPS-1 [12].

(Neuro) inflammation: Disorders in high energy phosphate metabolism in CRPS-1 patients [13], vascular leakage from macromolecules [14] and significantly higher concentrations of IL-6 and TNF alpha [15] and tryptase in fluid from artificially produced blisters in CRPS-1 patients, indicating mast cell activity [16], point to involvement of an inflammatory process in CRPS-1.

Arguments supporting the theory that neurogenic inflammation may play a role in triggering CRPS-1 are higher concentrations of bradykinin, neuropeptide Y, Calcitonin Gene-Related Peptide (CGRP) and vasoactive intestinal peptide in patients with CRPS-1 [17] and protein extravasation induced by substance P [18].

Tissue hypoxia: Some research findings point to tissue hypoxia in CRPS-1. Patients with the condition are more hyperalgesic to fluids of low pH than control subjects. This applies not only to the skin but also to the deeper somatic structures [19]. Patients with CRPS-1 have higher skin lactate levels than controls, suggesting a rise in anaerobic glycolysis as a consequence of chronic tissue hypoxia [20]. Capillary haemoglobin oxygenation of the skin is normal in control individuals, lower than normal in the affected limb following surgery, and lower than normal in both affected and control limbs of people with CRPS-1. This suggests skin hypoxia. Reduced blood circulation leading to under-nutrition of an affected limb may be a factor contributing to atrophy and ulceration [21].

Autonomic and motor dysfunctions: Autonomous symptoms are seen in 98% of CRPS-1 patients, but often change as the condition progresses [22]. Autonomic functions described in connection with CRPS-1 are vasomotor and sudomotor changes, changes at transmitter level and changes at receptor level. The vasoconstrictive response of thermoregulating skin circulation is slower in all phases of CRPS-1, suggesting sympathetic denervation, which can lead to hypersensitivity to catecholamines in vascular structures [23]. Motor dysfunction is common in patients with CRPS-1. Besides muscle weakness, patients can develop ‘neglect’ syndrome. The limb feels strange (cognitive neglect), and mental and visual attention is needed to move a limb (motor neglect) [24].

#### Pain exposure physical therapy

 Peripheral and central sensitization is a common feature in CRPS-1 as in other neuropathic pain syndromes [25]. Various studies have shown functional changes in patients with CRPS-1. Patients have altered central sensomotor response to tactile stimulation of the fingertip [26]. Referred sensations are a feature of CRPS-1, and this is evidence of central reorganization. Recent studies point to a crucial roleof the central nervous system (CNS) in the pathophysiology of CRPS-1 [27].

Not only the complex patterns of autonomic dysfunction, but also motor and sensory symptoms imply CNS alterations. Typically, active range of movement is restricted, whereas passive movement is often possible. Plastic CNS alterations might explain the complex sensory symptoms (e.g. glove-stocking sensory loss, 'foreign-hand’ sensation, mislocalization after tactile stimulation, impaired perceptual learning ability). Central changes show both shifts in sensory and motor cortex representation of the limbs in CRPS-1. This altered reorganisation seems to be associated with a pain perception as in phantom limb pain. Therefore, a lack of cortical re-reorganization could be an important factor for long term pain [28,29]. Patients with CRPS-1 show a significant reorganization of central motor circuits, with an increased activation of primary motor, parietal and supplementary motor cortices. In addition to fMRI studies, there are psychophysical studies showing that many patients with CRPS-1 suffer from cognitive and motor neglect-like symptoms. Summarizing these results, there is growing evidence that CNS alterations play an important role in the development and persistence of CRPS-1 [27].

Behavioural and psychological factors can exacerbate the pain and dysfunction in CRPS-1 and could help maintain the condition in some patients. Effective management of CRPS-1 requires that these aspects be addressed as part of an integrated treatment approach [30].

### Current evidence

#### Conventional therapy

Scientific argumentation for the physical therapy approach of CRPS-1 reveals a RCT with high level quality [6] indicating that physical therapy (in addition to medical treatment) has a clinically relevant positive effect on impairments of functions and pain. A systematic literature review [31] shows good to very good quality level II evidence that graded motor imagery is effective in reducing pain in adults with CRPS-1.

Analgesics are often used in clinical practice when treating patients with CRPS-1, and their use is described in various treatment protocols and guidelines [32-34]. The scientific support for their administration to patients with CRPS-1 is, however, very limited. Administration of standard analgesics appears to be based on experience in other fields. The administration sequence in Dutch practices is based on the Dutch national clinical guideline 2006, in accordance with the WHO’s pain ladder [10]. Oral administration of analgesics is followed by intravenous administration or peripheral blockade techniques [35-39].

Dimethylsulphoxide 50% ointment (DMSO) has a much greater beneficial impact on CRPS-1 symptoms than placebo according to several studies [40,41]. N-acetylcysteine, at a dose of 600 mg three times a day, was found to have a significantly better effect on primary cold CRPS-1 than DMSO ointment.

The anticonvulsant gabapentin causes a modest but significant reduction in neuropathic pain symptoms in CRPS-1 patients some weeks after the start of treatment [42,43].

No randomized control studies have been carried out on orally administered muscle relaxants to treat the motor symptoms of CRPS-1 [44]. Benzodiazepines and high doses of baclofen could have a positive effect.

Intrathecal baclofen therapy (ITB) is an invasive therapy and should be limited to carefully selected patients and, because of a high complication rate, should be conducted only by physicians with considerable experience in the implantation and care of intrathecal devices [45,46].

Muizelaar et al. investigated the effect of calcium-channel blockers in treating CRPS-1 [47]. They reported that they are most effective on CRPS-1 in the acute phase. However, the study is of moderate quality and size, and it is primarily descriptive. The outcomes were subjective, failing to describe the nature of the improvement in patients’ conditions.

Sympathetic nerve blockade is an accepted treatment option for CRPS-1 patients; for the upper limb blockage of the stellate ganglion or the thoracic sympathetic nerves, for the lower limb the lumbar sympathetic nerves, conventionally carried out at L2 and L3. However, no randomized studies have been carried out on CRPS-1 patients comparing the effect of sympathetic blockades performed under local anaesthetics with valid controls.

#### Pain exposure physical therapy

Regarding intensive physical therapy, including more aggressive or painful exercises (desensitization, aerobics, sports and loading), we found three retrospective analyses in children with CRPS indicating that in up to 92% of the included children, a complete recovery of symptoms occurred after intensive physical therapy [48-50]. Watson et al. show the effectiveness and safety of an active “stress loading” program in 41 patients with reflex sympathetic dystrophy, with improvement in pain, trophic and vasomotor changes, range of motion and grip strength [51].

Pain Exposure Physical Therapy has been evaluated in a large case series of 106 patients with chronic CRPS-1 [4]. Ninety-four percent of the patients showed a significantly improved function of the affected extremity, of which 49% experienced full functional recovery. In addition, a significant reduction in the number of symptoms of CRPS-1, such as pain (75% of patients improved), use of analgesics and aids, was registered. This case series suggests that PEPT is an effective and safe treatment for this group of patients, who are unresponsive to standard therapies.

We conducted a n = 1 study on 20 patients with acute CRPS-1 to evaluate the safety of this approach in acute CRPS-1 [3]. None of the patients in this case series experienced an exacerbation of CRPS-1 during the follow-up period of 3, 6 and 12 months. They showed a significant improvement in functions and pain.

### Aim

The aim of this study is to determine whether Pain Exposure Physical Therapy (PEPT) can improve functional outcomes in patients with CRPS-1.

## Methods/design

### Design of the study

This study is designed in accordance with the principles of a single-blinded, randomized clinical trial. After randomization to one of the two study groups, according to a pre-fixed scheme, baseline measurements (T0) are performed. Further measurements are taken during the course of the treatment at three months (T1) and at the end of the treatment at six months (T2). Follow up is at nine months (T3) after inclusion. Patients will undergo complete assessments at all time points.

Investigators performing the measurements will be blinded for the treatment group. Patients will be carefully instructed not to violate this blinding protocol.

This study is approved by the state regional ethical committee and is registered at http://www.clinicaltrials.gov with number NCT00817128 and at http://www.trialregister.nl with number NTR 2090.

### Setting

All patients will be screened, randomized and treated in our university hospital, a level 1 trauma centre in a rural area of The Netherlands. Approximately 150 patients with suspected CRPS-1 are referred to our multidisciplinary outpatient department annually.

### Study eligibility criteria

Hospitals and general practitioners in the region of our hospital will participate in recruitment of eligible patients. The diagnosis CRPS-1 has to be confirmed by Bruehl/IASP criteria (see below) and screening for eligibility is between three and twenty-four months after initial injury. This screening will take place at the outpatient department of the Radboud University Nijmegen Medical Centre. All patients, between 18 and 80 years old, with CRPS-1 of the upper or lower extremity will then undergo eligibility assessment.

All patients with possible underlying diagnoses which may cause the pain syndrome will be excluded. Impairments of the contra-lateral extremity, relapse of CRPS-1, pregnancy, lactation and prior sympathectomy of the affected extremity are criteria for exclusion.

All patients have to give written informed consent prior to participation in this study. An information letter will be provided, depending on the treatment group.

#### Modified research diagnostic criteria for CRPS

1. Continuing pain, disproportionate to any inciting event

2. At least one symptom in each of the four following categoriesa.Sensory: reports of hyperesthesiab.Vasomotor: reports of temperature asymmetry and/or skin colour changes and/or skin colour asymmetryc.Sudomotor/oedema: reports of oedema and/or sweating changes and/or sweating asymmetryd.Motor/trophic: reports of decreased range of motion and/or motor dysfunction (weakness, tremor, dystonia) and/or trophic changes (hair, nail, skin)

3. At least one sign in two or more of the following categoriesa.Sensory: evidence of hyperalgesia (to pinprick) and/or allodynia (to light touch)b.Vasomotor: evidence of temperature asymmetry and/or skin colour changes and/or skin colour asymmetryc.Sudomotor/oedema: evidence of oedema and/or sweating changes and/or sweating asymmetryd.Motor/trophic: evidence of decreased range of motion and/or motor dysfunction (weakness, tremor, dystonia) and/or trophic changes (hair, nail, skin)

4. The presence of an initiating noxious event, or a cause of immobilization

5. Symptoms cannot be explained by other diagnoses

### Sample size calculation

The sample size calculation is based on the following finding and expectation. The improvement in the Impairment level SumScore (ISS), restricted version [7,52], which is the primary outcome measure, for the conventional therapy group is 55% over one year [2]. For the PEPT-group it is estimated to be around 80% [4]. Given an alpha of 0.05 and a power of 80% for a one-sided Chi-square test, 62 patients are needed when using a 1:1 randomization, 31 in the PEPT-group and 31 in the conventional therapy group.

### Trial interventions

#### Conventional group

The conventional treatment of CRPS-1, based on the recent Dutch guideline [10], includes pharmacological interventions with analgesics, N-acetylcysteine, calcium channel blocker, ketanserine and dimethylsulphoxide for local application on the skin. In case of insufficient effect, sympathetic blockade, spinal cord stimulation and amputation may be considered. Following the Dutch guideline, patients in the conventional care group will also be referred to physical therapy to exercise the extremity in a pain contingent manner.

#### Experimental group

The experimental treatment is PEPT, a functional form of physical therapy combined with a cognitive-behavioural form of treatment. It consists of a maximum of five sessions of each 40 min. Analgesics are contra-indicated, the experienced pain will not be treated.

### Patient recruitment

Diagnosis and treatment of CRPS-1 is performed by the multidisciplinary CRPS outpatient team, including a surgeon, a rehabilitation physician, a physical therapist, an anaesthesiologist and a research nurse. Around 150 new adult patients are visiting this outpatient clinic each year of which 23% is diagnosed having CRPS-1 (confirmed by Bruehl/IASP criteria) between 3 and 24 months after initial injury and without underlying diagnoses which may be responsible for the pain syndrome [53]. This means that around 40 patients with CRPS-1 are diagnosed each year, who can be randomized accordingly. To increase the amount of eligible patients, the county hospitals as well as general practitioners in Nijmegen and surroundings will be asked to pre-screen and send patients with possible CRPS-1 to our outpatient department for screening and probable enrolment into the study. It is expected that this strategy will increase the amount of eligible patients. All these eligible patients will be screened by the multidisciplinary CRPS outpatient team prior to inclusion. Both the conventional treatment and PEPT will be executed in the Radboud University Nijmegen Medical Centre, with blinding of the observer during all measurements.

### Time schedule

Patients will be screened and randomized from January 2009 until June 2011. A mean of 4 patients are planned to enrol each month once the study reaches full adhesion. After complete follow-up, it will be finished in the spring of 2012. The final report after data analysis will be expected at the end of 2012.

### Randomization

Patients will be pre-screened, based on the referral letter sent by the county hospital or general practitioner, during a multi-disciplinary session of our team. In cases of potentially positive CRPS-1 in accordance with Bruehl/IASP criteria, these patients are invited to undergo thorough screening and multidisciplinary assessment by the same team. Patients with manifest CRPS-1 in accordance with the research diagnostic criteria of Bruehl/IASP, judged unanimously by the team, are asked to participate in the study. They are informed about the study by the research nurse and if they are interested, one week of consideration is scheduled. During this week, an accelerometer (PAM, physical activity monitor) is mounted on the affected wrist (CRPS-1 in the upper extremity) or affected ankle (CRPS-1 in the lower extremity) to measure the intensity of activity in T0. After this one week of consideration, a custom made computed randomization program is used to blindly allocate patients to one of the two treatment groups. The randomization program is made by an investigator with no clinical involvement in the trial.

Every patient who will be screened will specifically be informed about the possibility to change treatment group at any time after randomization, but preferably after having finished the initial allocated treatment. Once the computer randomization has been completed, patients who are allocated to the conventional care group are scheduled for their first treatment visit at the outpatient department of anaesthesiology within two weeks. Patients who are allocated to the PEPT group are scheduled for their first treatment at the outpatient department of rehabilitation within two weeks.

Each randomization package contains the following forms:

· Patient identification tabs

· Checklist hospital contacts

· Outpatient chart

· All written correspondence

· Travel compensation forms

· Randomization form

· Informed consent

· Measurement sets (mentioned under “outcome parameters”) for all measure moments

· Diaries (physical activity, pain score and medication during seven days) for all measure moments

· Cost lists (e.g. doctor’s visits, medical costs) for all measure moments

· Questionnaires (mentioned under “outcome parameters”) for all measure moments

· PAM scoring list

· PAM result list

### Blinding procedure

All measurements taken in both treatment groups during the course of this study are carried out by an experienced research nurse who underwent extensive measurement instructions, training and supervision before she was considered qualified to perform all measurements. She is blinded for the treatment group in all patients. In order to maintain this blindness, we thoroughly instruct all patients not to violate her blindness. To further guarantee her blindness, we instruct her to put a small amount of menthol ointment on her upper lip during each measurement session. This prevents her from smelling the garlic odour of DMSO ointment, which is part of the conventional treatment.

### Outcome parameters

#### Primary outcome measures

The primary outcome measure is the Impairment level SumScore (ISS), restricted version [7,52]. The ISS, restricted version, consists of three measurement parameters (pain, active range of motion and temperature) and four measurement instruments (Visual Analogue Scale for Pain (VAS-P) [54], McGill Pain Questionnaire Dutch Language Version (MPQ-DLV) [55], goniometry of mobility of joints [56], and skin temperature) [57] and has a range of 4 to 40 points. Beside the ISS, restricted version, its individual components will be evaluated [52].

#### Secondary outcome measures

1. The Disability of the Arm, Shoulder and Hand (DASH), a questionnaire which maps both functions and daily activities of the upper limb [58,59]. The Lower-Limb Tasks Questionnaire (LLTQ) of McNair [60], a questionnaire which focuses on physical tasks related to function of the lower limb.

2. The fear-avoidance beliefs questionnaire (FABQ) [61], a statement list regarding the perception on pain and physical activities

3. The level of participation as determined by the questionnaire SF-36 [62], which measures the patient’s point of view regarding health

4. At function level, the muscle force measurements, as derived from both hands and feet by a handheld dynamometer (MicroFET) [63,64].

5. At level of activity limitations, for the legs the 10 m walking test [65] (which measures the time walking a certain distance) and the Timed Up and Go test [66,67] (which measures the time from rising from a chair, walking a restricted distance to sitting down again).

6. Compliance and adherence: adherence to long-term therapy is defined by the World Health Organization as the extent to which a person’s behaviour corresponds with agreed recommendations from a healthcare provider [68]. Physical activities like walking and exercises, combined with an adequate use of medication, aids and appliances, are essential to guarantee success in the present study on patients with CRPS-1. However, little is known about the level of physical activity, the intensity of exercises and the adherence of these patients to recommendations from the physician and physical therapist. Therefore, an extensive assessment will be performed by interview (about activities such as changing and maintaining body positions, carrying, handling and moving objects, walking and moving, self-care and household tasks, and exercises), questionnaires (the Seven Days Physical Activity Recall questionnaire (PAR) [69,70], the International Physical Activity Questionnaire (IPAQ) [71], the Pain Catastrophizing Scale (PCS; a questionnaire regarding the perception of pain) [72] and the Pain Disability Index (PDI; a list measuring the influence of pain complaints on daily life) [73]) and an accelerometer (PAM) [74] to monitor compliance and adherence. The PAM will be used as a control device for over- or under-reporting of physical activity via questionnaires (PAR, IPAQ). Patients will be instructed to wear the PAM from the moment they go out of bed in the morning until the moment they go to bed at the end of the day. Scores for self-reported physical activity will be combined with PAM-scores to validate self-reported physical activity. Self-reported adherence with PEPT will be combined with observations of physicians and physical therapists.

7. Quality of life will be measured with the EuroQol (EQ-5D) [75]; this is on behalf of the economic evaluation

8. Adverse reactions will be monitored throughout the trial using standardized Serious Adverse Event forms, specifically regarding exacerbations of CRPS-1 signs and symptoms leading to medical consultation.

### Economic analysis

This study investigates the potential efficiency of PEPT versus conventional treatment in patients with CRPS-1 from a societal perspective. The economic evaluation is based on the general principles of a cost-effectiveness analysis. Primary outcome measures for the economic evaluation are costs and quality adjusted life years (QALYs). The ratio cost per QALY gained (ICER, incremental cost effectiveness ratio) will be computed and uncertainty will be determined using the bootstrap method or Fieller method. Finally, a cost-effectiveness acceptability curve will be derived that is able to evaluate efficiency by using different thresholds (WTP, willingness to pay) for a QALY. The impact of uncertainty surrounding deterministic parameters (for example cost-prices) on the ICER will be explored using one-way sensitivity analyses on the range of extremes. The economic evaluation is being done alongside the clinical trial and consequently adheres to the earlier presented design and measurement points.

#### Patient outcome analysis

The effect analysis adheres to the design of a randomized controlled trial, relevant for the economic evaluation quality of life. Quality adjusted life years (QALYs) will be computed (using the trapezium rule) in order to perform a cost-utility analysis for the two alternative strategies. For the overall quantification of health status as a single index (utilities) we use the standard EQ-5D classification system developed by the EuroQol Group. The EQ-5D is one of the three widely used multi-attribute systems available to determine health states preferences (utilities). We chose the EQ-5D because 1) the five domains of the EQ-5D reflect aspects that are thought to be important for the population under consideration, 2) the system is relatively simple to administer, 3) the sensitivity of the instrument proved to be satisfactory, and 4) a reasonable sound algorithm has been published to compute utilities.

#### Cost analysis

The cost analysis exists of two main parts. First, on patient level, volumes of care will be measured prospectively using standardized Case Report Forms (CRF) and patient-based diaries. Per treatment group, full cost-prices will be determined using activity based costing. Activities in both production processes are outpatient visits and physical therapy. Medication costs constitute of analgesics, DMSO 50%, n-acetylcysteine, gabapentine, carbamazepine, amitriptylin, nortriptylin, baclofen, diazepam, clonazepam and sympathetic blocks. Productivity losses for patients will be estimated using an interview on a 2 months recall basis by the researcher. The frictioncost-method will be applied. Also travel time to therapy or outpatient clinic and related costs will be considered, on the basis of 2 months recall.

The second part of the cost analysis consists of determining the cost prices for each volume of consumption in order to use these for multiplying the volumes registered for each participating patient. The Dutch guidelines for cost analyses will be used [76]. For units of care/resources where no guideline or standard prices are available, real cost prices will be determined.

The potential effects on medical costs are expected to be approximately €2802,75 per treatment in conventional care and around €1008,50 per treatment in PEPT. Costs based on social participation and work are not included in these prices (see Table 1).

**Table 1:** Medical costs

	**Conventional therapy (CBO 2006)**	**Euro**	**Experimental therapy (PEPT)**	**Euro**
**Medical costs**				
Outpatient department (first visit)	1 time	45	1 time	45
Outpatient department (succeeding visits)	6 times	192	1 time	32
Physical therapy (first session)	1 time	65	2 (1 x 2 therapists)	130
Physical therapy (succeeding sessions)	25 times	1218.75	10 (5 x 2 therapists)	487.50
Travel expenses	33 times	740	14 times	314
**Other medical costs**				
Analgesics in accordance with WHO-standard (until step 2)	6 months NSAID 3 months tramadol Paracetamol	150 90	n.a.	n.a.
DMSO 50%	3 months	60	n.a.	n.a.
n-acetylcysteïne Gabapentine Carbamazepine Amitriptyline/Nortriptyline	6 months á €80 6 months à €15 6 months à €5	200	n.a.	n.a.
**Miscellaneous**				
Baclofen; Diazepam; Clonazepam	6 months à €13 x 0.1	7	n.a.	n.a.
Vasoactive agents	6 months à €9 x 0.4	10		
Sympathetic block (per treatment)	€125 x 0.2	25	n.a.	n.a.
**Total costs per patient per year**		** 2802.75**		**1008.50**

### Statistical analysis

All measured data will be assembled in a computer database and analyzed using SPSS 17.0. For all outcome variables, “effects” will be calculated at all evaluation time points during as well as after treatment, by taking values at T0 as reference values. Outcomes for each group will be plotted graphically in time to study their patterns. Linear mixed models will be used to examine possible differences over the course of time and to find out if these differences between treatment groups can be considered as statistical significant. For the level of significance alpha = 0.05 will be used. As the difference between groups in ISS, restricted version, is expected to be around 25%, we take this as a minimal clinically important difference (MCID) in effect between groups between T0 (intake) and T3 (follow-up) for the primary outcomes.

For the secondary outcomes, even as for cost differences, explorative tests will be used (Wilcoxon). Two analyses will be performed: an intention-to-treat analysis (ITT) and a per-protocol analysis (PP). In the ITT analysis, outcomes of all the participants will be used for the group they are originally assigned to. In the PP analysis, outcomes of protocol violators will be ignored.

## Discussion

Clinical trials on the treatment of patients with CRPS-1 are scarce due to deficiency and controversy in objective diagnostic criteria and underlying pathophysiology. Most clinicians support an approach of multiple interventions at different dimensions, customized to the individual patient. Therefore, only few patients can be identified with a homogenous clinical presentation for whom a therapeutic approach can be standardized. Intervention trials in patients with CRPS-1 have therefore typically been focused on a single item of this approach. This makes the results of individual trials difficult to interpret and the comparison of multiple trials impossible. One of the most important efforts to improve the level of evidence for treatments in this field is to equalize diagnostic criteria. At present, the Bruehl/IASP criteria are the international standard to diagnose a patient with suspected CRPS-1, not only for research applications but also in clinical practice. In our outpatient department for CRPS-1 patients, we use these criteria without restraints ever since 2004. Clinical studies from The Netherlands have been highly respected internationally due to the large amounts of patients that are treated in our country. It has to be confirmed that the diagnostic criteria according to Veldman, which have been used nationwide, are no longer valid. More than 75% of patients who are referred with positive Veldman criteria do not have CRPS-1 according to the Bruehl/IASP criteria [53].

Due to the evolvement in diagnosing CRPS-1, it is possible to recruit a homogenous patient group, which is eligible for solid prognostic studies such as the current one.

The new and promising approach in the treatment of CRPS-1, PEPT, has proven to be safe and possibly effective, and is now ready for clinical evaluation.

This is the first randomized controlled study with single blinding that has ever been planned in patients with CRPS-1 and does not focus on one single item but compares treatment strategies based on completely different pathophysiological and cognitive theories.

## Abbreviations

CRPS-1: Complex Regional Pain Syndrome type 1; PEPT: Pain Exposure Physical Therapy; IASP: International Association for the Study of Pain; WHO: World Health Organization; DMSO: Dimethylsulphoxide 50% ointment; CNS: Central nervous system; fMRI: Functional Magnetic Resonance Imaging; ISS: Impairment level SumScore; PAM: Physical activity monitor; QALY: Quality adjusted life year.

## Competing interests

Financial competing interests.

The authors declare that they have no financial competing interests.

Non-financial competing interests.

The authors declare that they have no non-financial competing interests.

## Authors’ contributions

KB largely rewrote the manuscript after critically revising it, and finished the manuscript. RO contributed substantially to conception and design and by revising it critically for important intellectual content. RvD assisted in study design and drafted the parts on conventional therapy. FK assisted in study design and drafted the parts on the experimental treatments. HS assisted in study design and drafted the parts on secondary outcome parameters. GJvdW assisted in study design and drafted the parts on power calculation and randomization. EA assisted in study design and drafted the parts on economical and cost analysis. HG drafted the parts on statistical analysis. HvdM conceived of the study, and participated in its design and coordination and helped to draft the manuscript. JPF originally drafted the manuscript in accordance with the awarded research grant from the Dutch Government (ZonMw). All authors read and approved the final manuscript.

## Authors’ information

KB is currently a medical student at the Radboud University Nijmegen.

RO started his education in physical therapy (PT) at the Sint Maartenskliniek, Nijmegen, The Netherlands in 1963. He received his bachelor degree PT in 1966 and his degree in Manual Physical therapy (MPT) in 1972. He studied rehabilitation sciences (MSc) at the Free University of Brussels, Belgium which he finished in 1984 cum laude. Between 1984–1988 he did his PhD at the Radboud University Nijmegen, The Netherlands. Between 1968–1982 he was head of the department of physical therapy in general hospitals and teacher at the School of Physical Therapy ‘West Brabant’, Breda, The Netherlands. Between 1988–2007, he became research director of the Dutch Institute of Allied health Care, Amersfoort, The Netherlands. Between 1989–2000 he was full professor Manual Therapy at the Free university of Brussels, Belgium. From 2000 until his retirement in 2008, he was full professor in Allied Health Sciences at the Radboud University Nijmegen, The Netherlands, with a focus on the quality of allied health care and development, implementation and evaluation of clinical guidelines. He has a long-standing practical experience with regards to treatment of patients with Complex Regional Pain Syndrome (CRPS). He has published about 250 articles in national and international journals. He is member of various national and international physical therapy organizations and reviewer of national and international journals.

RvD is anaesthesiologist at the Pain Centre of the Radboud University Nijmegen Medical Centre, The Netherlands since 1988. He participates in all activities in an Academic Multidisciplinary Pain Centre for acute pain, chronic pain, pain in cancer and palliative care. Specific expertise in invasive pain treatment, neuromodulation for pain relief. He is Chairman of the Dutch Society for Neuromodulation and former Chairman of Pain Section of the Dutch Society for Anaesthesiology.

FK is working as a physical therapist since 33 years in his own practice and at the Department of Physical Therapy of the Radboud University Nijmegen Medical Centre. Specific expertise in treatment of patients with chronic pain and Complex Regional Pain Syndrome (CRPS). He is also working as a Quality of Care employee at the Department of Physical Therapy.

HS has been working as a clinical psychologist in pain medicine since 1990 and is working at the Academic Centre of Pain and Palliative Care of the Radboud University Nijmegen Medical Centre. He successfully finished his PhD thesis in 2008, titled Psychological factors in chronic pain: from predictors to implementation. In 2002 he wrote a book titled The psychologist as pain specialist. He is specialized in diagnosing and treating chronic pain within tailored treatment designs and in translating scientific knowledge into clinical practice for doctors, psychologists, physical therapists and nurses.

GJvdW is professor of Health Technology Assessment at the Radboud University Nijmegen Medical Centre and professor of Interactive Health Technology Assessment at the Athena Institute, Free University Medical Centre, Amsterdam. He is a member of the Standing Committee on Medical Technology Assessment of the Health Council of The Netherlands and member of the National Appraisal Committee at the National Health Insurance Board. He serves on the Editorial Board of the International Journal of Technology Assessment in Health Care and is a member of the International Network of Agencies for Health Technology Assessment (INAHTA).

EA studied graduated as an economist in 1990. Then a professional career was started as a university lecturer at Maastricht University, faculty of Economics. In 1997, he successfully finished his PhD thesis (promoter, prof. dr. G. Kootstra, surgeon). Earlier, in 1996, he started as an assistant professor at the department of Health, Organization, Policy, and Economics (BEOZ) of the faculty of Health Sciences, Maastricht University. In the beginning of 2001 he started as a senior consultant at the Ministry of Economic Affairs in The Hague, which he left late 2001 for an assistant professorship at the Radboud University Nijmegen Medical Centre. Here, he joined the department of Medical Technology Assessment of which he became deputy head in 2004. Presently the department is merged into the department of Epidemiology, Biostatistics and HTA. Several publications about economical aspects of health care are from his hand.

HG studied Biology and Biophysics at the Radboud University Nijmegen (Msc; 1985). In 1986, after additional training in information technology, he joined the department of Epidemiology as a scientific programmer. He specialized in methodological and statistical support of several PhD projects. He also lectured in statistical methods for Biomedical students. From 2005 on, he specialized in Health Technology Assessment. Nowadays, he participates in several research projects of major departments at the Radboud University Nijmegen Medical Centre as a methodological and statistical consultant.

HvdM is consultant at the Department of Rehabilitation Medicine at the Radboud University Nijmegen Medical Centre, The Netherlands. He worked at the department of neurosurgery Erasmus UMC Rotterdam and AMC Amsterdam, both in The Netherlands. He worked as post-doc at the Institute for Brainresearch Zurich, Switzerland and as house officer neurology both at the University Clinic Balgrist Zurich, Switzerland and at the NRZ Greifswald in Germany. He was trained as rehabilitation physician at the Sint Maartenskliniek in Nijmegen, The Netherlands. Since 2003, he is associate professor at the Department of Rehabilitation Medicine at the Radboud University Nijmegen Medical Centre, The Netherlands with specialization in care, research and education in management of spasticity in children with cerebral palsy, acute management and rehabilitation of spinal cord injury, development of innovative orthotics and prosthesis for trauma and orthopaedic rehabilitation, early intensive physical and cognitive rehabilitation of patients with critical illness with application of virtual reality devices (X-VR-D) and experimental management and rehabilitation of patients with CRPS-1. He cooperates in the multidisciplinary outpatient department for patients with CRPS since 2004.

JPF is consultant (general and orthopaedic) trauma surgeon at the Department of Surgery from the Radboud University Nijmegen Medical Centre in The Netherlands. He graduated from The University of Utrecht Medical School, The Netherlands in 1990 with a specialization in General Surgery in 1996 and a subspecialization in Trauma Surgery in 2001 at the Free University Medical Centre in Amsterdam, The Netherlands. In the same year he defended his thesis successfully. In 2009, he graduated in clinical epidemiology at the Radboud University Nijmegen, The Netherlands. In 2004, he adopted the outpatient department for CRPS patients from his famous predecessor professor dr. RJA Goris, who retired at that time.

## Pre-publication history

The pre-publication history for this paper can be accessed here:

http://www.biomedcentral.com/1471-2474/13/58/prepub
